# Supplementary material for: Comparative Diagnostic Accuracy of the STANDARD M10 Assay for the Molecular Diagnosis of SARS-CoV-2 in the Point-of-Care and Critical Care Settings
Source: J Clin Med. 2022 Apr 27;11(9):2465. doi: 10.3390/jcm11092465 (PMC9105343; doi:10.3390/jcm11092465)
Supplement: Supplementary file 1 [file jcm-11-02465-s001.zip › jcm-1691358-supplementary.pdf]

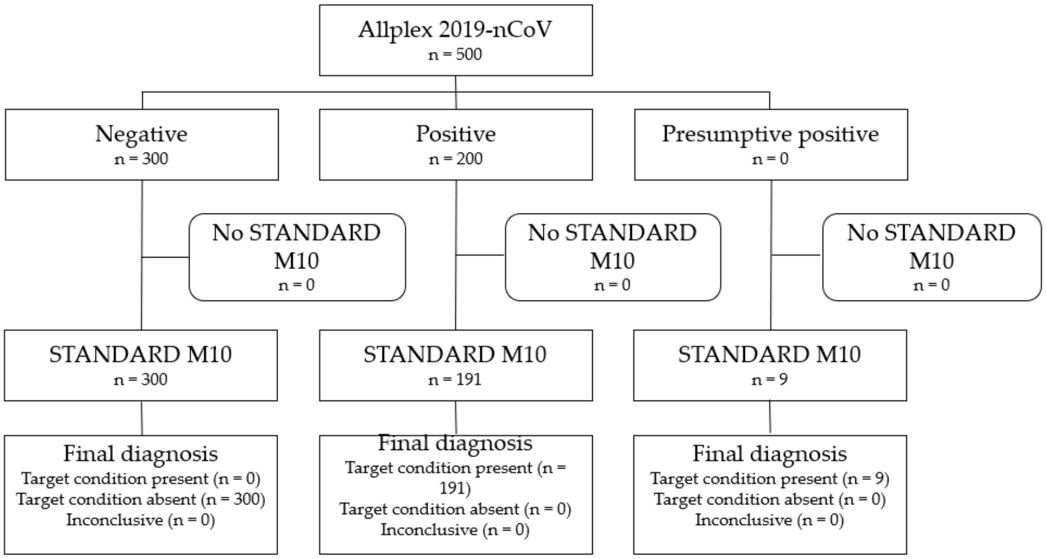

Figure S1. STARD (Standards for Reporting of Diagnostic Accuracy Studies) flowchart.

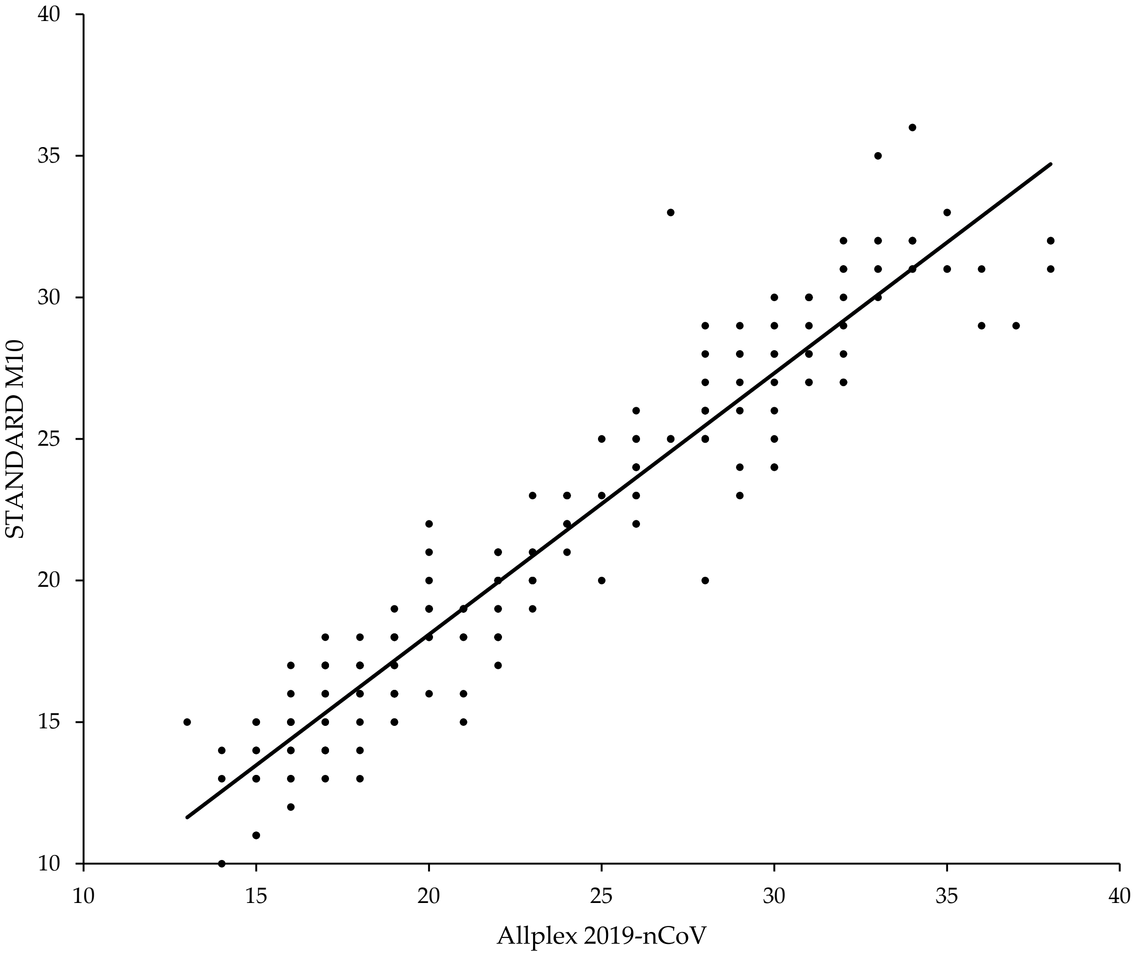

Figure S2. Correlation between E gene cycle thresholds provided by the STANDARD M10 and Allplex 2019-nCoV assays.

Table S1. STARD (Standards for Reporting of Diagnostic Accuracy Studies) checklist.

Section & Topic	No	Item	Reported in Section (§)
**TITLE OR ABSTRACT**			
	**1**	Identification as a study of diagnostic accuracy using at least one measure of accuracy (such as sensitivity, specificity, predictive values, or AUC)	Title (NA)
**ABSTRACT**			
	**2**	Structured summary of study design, methods, results, and conclusions (for specific guidance, see STARD for Abstracts)	Abstract (NA)
**INTRODUCTION**			
	**3**	Scientific and clinical background, including the intended use and clinical role of the index test	1 (1–4)
	**4**	Study objectives and hypotheses	1 (4)
**METHODS**			
Study design	**5**	Whether data collection was planned before the index test and reference standard were performed (prospective study) or after (retrospective study)	2.1 (4)
Participants	**6**	Eligibility criteria	2.1 (3)
	**7**	On what basis potentially eligible participants were identified (such as symptoms, results from previous tests, inclusion in registry)	2.1 (3)
	**8**	Where and when potentially eligible participants were identified (setting, location and dates)	2.1 (2–4)
	**9**	Whether participants formed a consecutive, random or convenience series	2.1 (4)
Test methods	**10a**	Index test, in sufficient detail to allow replication	2.2 (1–3)
	**10b**	Reference standard, in sufficient detail to allow replication	2.3 (1–3)
	**11**	Rationale for choosing the reference standard (if alternatives exist)	2.3 (1)
	**12a**	Definition of and rationale for test positivity cut-offs or result categories of the index test, distinguishing pre-specified from exploratory	2.3 (1)
	**12b**	Definition of and rationale for test positivity cut-offs or result categories of the reference standard, distinguishing pre-specified from exploratory	2.3 (1)
	**13a**	Whether clinical information and reference standard results were available<br>to the performers/readers of the index test	2.1 (3)
	**13b**	Whether clinical information and index test results were available<br>to the assessors of the reference standard	2.1 (3)
Analysis	**14**	Methods for estimating or comparing measures of diagnostic accuracy	2.4 (1)
	**15**	How indeterminate index test or reference standard results were handled	2.2 (1)
	**16**	How missing data on the index test and reference standard were handled	2.4 (1)

	**17**	Any analyses of variability in diagnostic accuracy, distinguishing pre-specified from exploratory	2.4 (1)
	**18**	Intended sample size and how it was determined	2.4 (3)
**RESULTS**			
Participants	**19**	Flow of participants, using a diagram	Figure S1
	**20**	Baseline demographic and clinical characteristics of participants	3 (1,2)
	**21a**	Distribution of severity of disease in those with the target condition	NA
	**21b**	Distribution of alternative diagnoses in those without the target condition	NA
	**22**	Time interval and any clinical interventions between index test and reference standard	2.1 (4)
Test results	**23**	Cross tabulation of the index test results (or their distribution)<br>by the results of the reference standard	3 (Table 1)
	**24**	Estimates of diagnostic accuracy and their precision (such as 95% confidence intervals)	3 (Table 3)
	**25**	Any adverse events from performing the index test or the reference standard	NA
**DISCUSSION**			
	**26**	Study limitations, including sources of potential bias, statistical uncertainty, and generalisability	4 (6)
	**27**	Implications for practice, including the intended use and clinical role of the index test	4 (1–5)
**OTHER INFORMATION**			
	**28**	Registration number and name of registry	NA
	**29**	Where the full study protocol can be accessed	NA
	**30**	Sources of funding and other support; role of funders	Funding (1)

Table S2. Limit of detection (LOD) for eNAT samples, by gene target and SARS-CoV-2 concentration.

Gene Target	% (*n* Detected/*N* Tested) at a Given SARS-CoV-2 Concentration, Copies/ml						95% LOD, copies/ml
	**600**	**300**	**150**	**75**	**37.5**	**18.75**	
≥ 1 gene	100 (6/6)	100 (6/6)	80 (4/5)	67 (4/6)	50 (3/6)	17 (1/6)	235
ORF1ab	83 (5/6)	83 (5/6)	80 (4/5)	50 (3/6)	33 (2/6)	17 (1/6)	1,056
E	100 (6/6)	100 (6/6)	80 (4/5)	33 (2/6)	50 (3/6)	17 (1/6)	326
